# Oily fish and raw vegetable consumption can decrease the risk of AQP4-positive neuromyelitis optica spectrum disorders: a Mendelian-randomization study

**DOI:** 10.1038/s41598-023-36372-1

**Published:** 2023-06-09

**Authors:** Shengnan Wang, Lin Pan, Rui Wu, Yanqing Shao, Mengru Xue, Hao Zhu, Wanwan Min, Xiangyu Zheng, Yekun Liang, Mingqin Zhu

**Affiliations:** 1 Department of Neurology, Neuroscience Center, The First Hospital of Jilin University, Changchun, China; 2 Clinical College, Jilin University, Changchun, China; 3 Department of Hepatology, The First Hospital of Jilin University, Changchun, China; 4 Department of Cardiology, The First Hospital of Jilin University, Changchun, China

**Keywords:** Demyelinating diseases, Immunogenetics, Nutrition

## Abstract

Neuromyelitis optica spectrum disorders (NMOSD) are severe inflammatory disorders of the central nervous system targeting aquaporin‐4 (AQP4). The risk factors for NMOSD remain to be determined, though they may be related to diet and nutrition. This study aimed to explore the possibility of a causal relationship between specific food intake and AQP4-positive NMOSD risk. The study followed a two-sample Mendelian randomization (MR) design. Genetic instruments and self-reported information on the intake of 29 types of food were obtained from a genome-wide association study (GWAS) on 445,779 UK Biobank participants. A total of 132 individuals with AQP4-positive NMOSD and 784 controls from this GWAS were included in our study. The associations were evaluated using inverse-variance-weighted meta-analysis, weighted-median analysis, and MR-Egger regression. A high consumption of oily fish and raw vegetables was associated with a decreased risk of AQP4-positive NMOSD (odds ratio [OR] = 1.78 × 10^−16^, 95% confidence interval [CI] = 2.60 × 10^−25^–1.22 × 10^−7^, *p* = 0.001; OR = 5.28 × 10^−6^, 95% CI = 4.67 × 10^−11^–0.598, *p* = 0.041, respectively). The results were consistent in the sensitivity analyses, and no evidence of directional pleiotropy was observed. Our study provides useful implications for the development of AQP4-positive NMOSD prevention strategies. Further research is needed to determine the exact causal relationship and mechanisms underlying the association between specific food intake and AQP4-positive NMOSD.

## Introduction

Neuromyelitis optica spectrum disorders (NMOSD) are a group of severe autoimmune demyelinating diseases of the central nervous system (CNS), characterized by optic neuritis and longitudinally extensive myelitis (LETM)^1^. The presence of autoantibodies against aquaporin 4 (AQP4) is a hallmark of NMOSD^2^, occurring in 80% of patients with this disease, which is also considered an autoimmune astrocytomosis^3^. NMOSD mostly affect young adults, particularly women^4^. NMOSD are caused by inflammation, and patients with these disorders are prone to peripheral and CNS inflammation caused by cytokines, particularly those produced by T helper (Th)2 and Th17 lymphocytes^5^.

Several risk factors have been associated with NMOSD, including environmental and genetic factors^4,6^. A thorough investigation on these associations has yet to be conducted; however, several environmental risk factors are known, such as specific dietary patterns in both sexes, and in women, history of abortion or trauma, low body mass index (BMI), and low physical activity levels^7,8^.

Different dietary patterns determine the variance in the gastrointestinal microbiome^6^. According to previous studies, a high sugar intake results in dysregulation and decrease in microbiota diversity^9,10^. A dysbiosis of gut microbiota can lead to systemic and neuro-inflammation by increasing the levels of interleukin (IL)-6, tumor necrosis factor (TNF)-α, and IL-1^6,11,12^. Additionally, a retrospective study showed that diets with high inflammatory potential are associated with an increased risk of NMOSD^13^.

Dietary risk factors associated with NMOSD progression have been identified in previous studies^6,14^, though the evidence was insufficient to establish causal roles. A cross-sectional study was conducted to determine the type of diet mostly associated with the incidence of NMOSD. However, these observations may have been confounded by unidentified factors, and the causality of the associations was not supported^15^. Randomized controlled trials (RCTs) are the gold standard to determine a causal relationship^16,17^, though they are challenging to implement due to ethical constraints. It is often impossible to link specific nutritional interventions to disease outcomes in long-term RCTs because of the difficulty in selecting appropriate control groups and blinding participants and researchers^18^. These limitations can be overcome using Mendelian randomization (MR).

A growing number of studies have used MR to examine the possible causal role of modifiable exposures on the incidence of several diseases. MR is a statistical framework used to estimate the effects of exposure using genetic variants^19,20^. In the MR model, different allelic compositions result in different exposures throughout life, as genetic variants alter or mimic nutritional exposures (such as circulating micronutrients, macronutrient intake, and dietary patterns). Therefore, these variants may also contribute to disease risk^21^. MR can help overcome the limitations typical of observational studies, such as residual confounding, reverse causation, and recall bias^22^.

Single-nucleotide polymorphisms (SNPs) associated with dietary patterns and macronutrient intake can be used in MR analyses. Since SNPs cannot be modified, they are less susceptible to reverse causality due to Mendel’s second law, and thus MR using them is less likely to be affected by confounding factors and less prone to random or systematic measurement errors than other types of analysis. A key benefit of MR is that it allows to estimate the causal effect of an exposure on the occurrence of a disease using statistical analyses to mitigate the biases encountered in observational nutritional epidemiology.

A better understanding of how different types of foods affect the risk of NMOSD may help develop more effective prediction, treatment, and prevention strategies. The purpose of this study was to assess the causal relationship between dietary patterns and NMOSD using the MR method^16^.

## Materials and methods

### Genetic association between food intake and AQP4-positive NMOSD

**Figure 1:**
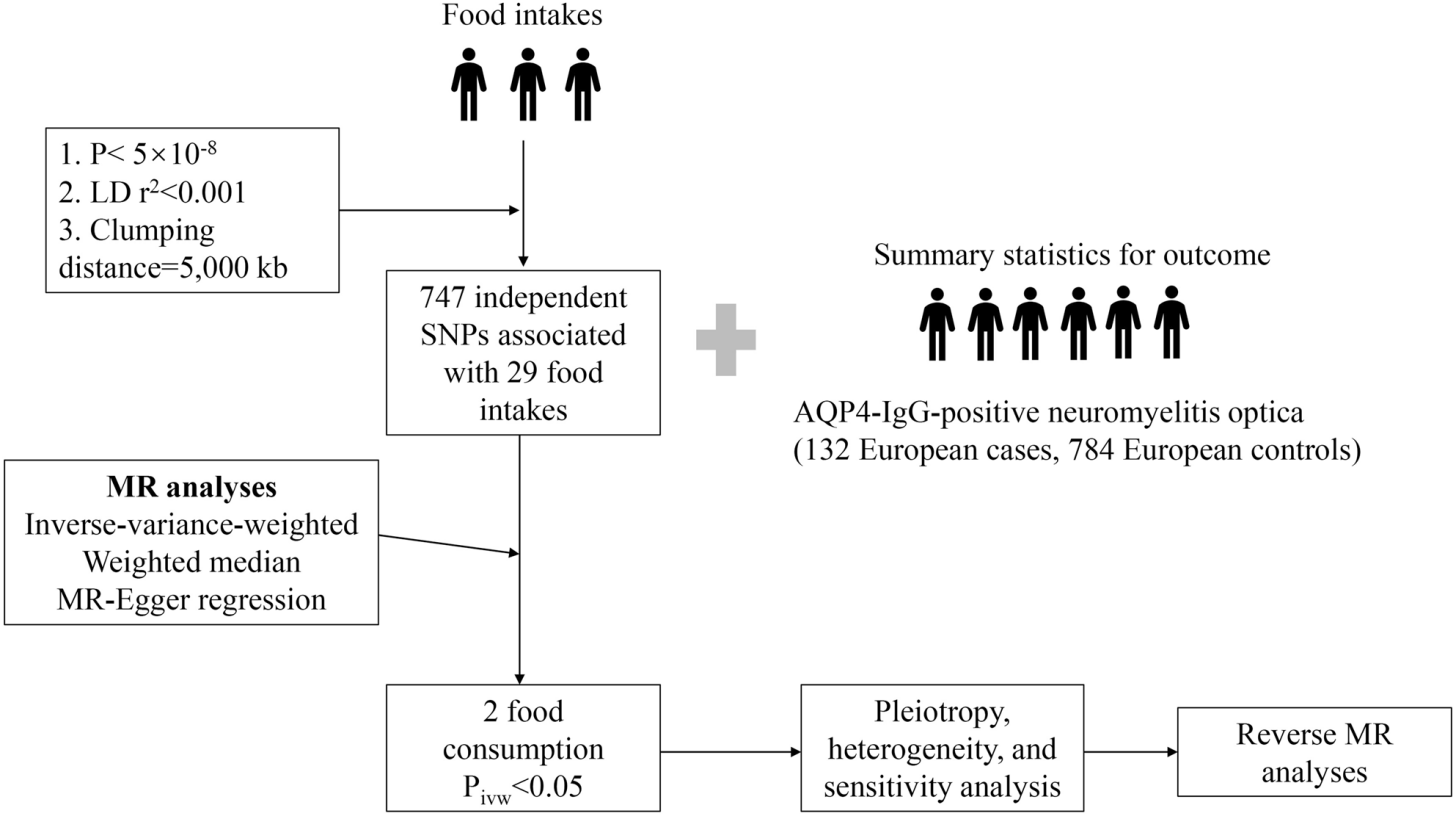
Flowchart of the MR analysis in this study. AQP4: aquaporin 4; SNPs: single-nucleotide polymorphisms; MR analysis: Mendelian randomization analysis.

An overview of the study design is shown in a flowchart (Fig. 1). A large recent genome-wide association study (GWAS) has identified several SNPs associated with food intake and patterns. Full GWAS summary statistics are available at https://www.ebi.ac.uk/gwas/home. We examined 29 lifestyle and dietary factors, including consumption of alcohol, coffee, fish, fruit, vegetables, and beef. A summary of each type of exposure is provided in Table 1. Genetic association data for NMOSD were drawn from a GWAS meta-analysis of this disease (GCST006937); our study included 132 AQP4-positive patients and 784 controls. The presence of NMOSD was determined using the 2006 diagnostic criteria, which include optic neuritis, transverse myelitis, and two of the following three supportive elements: (1) longitudinally extensive lesions (≥ 3 vertebral segments in length); (2) magnetic resonance imaging of the brain with findings not consistent with multiple sclerosis; and (3) AQP4-IgG antibody seropositivity^1^.

**Table 1:** Descriptive information of 38 lifestyle and dietary factors.

Phenotype	Sample size	GWAS ID	Pubmed ID
Beef consumption	241,092 European	GCST90096901	35,653,391
Beer or cider consumption	168,237 European	GCST90096902	35,653,391
Spread on bread consumption	445,506 European	GCST90096903	35,653,391
Bread consumption	438,853 European	GCST90096904	35,653,391
Champagne or white wine consumption	175,549 European	GCST90096905	35,653,391
Cheese consumption	365,842 European	GCST90096906	35,653,391
Cooked vegetables consumption	435,417 European	GCST90096907	35,653,391
Decaffeinated coffee consumption	62,072 European	GCST90096908	35,653,391
Dried fruit consumption	409,125 European	GCST90096909	35,653,391
Drink temperature	444,097 European	GCST90096910	35,653,391
Fortified wine consumption	31,836 European	GCST90096911	35,653,391
Fresh fruit consumption	433,186 European	GCST90096912	35,653,391
Ground coffee consumption	72,276 European	GCST90096913	35,653,391
Instant coffee consumption	180,764 European	GCST90096914	35,653,391
Lamb consumption	189,984 European	GCST90096915	35,653,391
Percentage fat in milk consumption	411,503 European	GCST90096916	35,653,391
Non-oily fish consumption	318,136 European	GCST90096917	35,653,391
Oily fish consumption	297,881 European	GCST90096918	35,653,391
Pork consumption	187,202 European	GCST90096919	35,653,391
Poultry consumption	399,716 European	GCST90096920	35,653,391
Processed meat consumption	312,220 European	GCST90096921	35,653,391
Red wine consumption	211,628 European	GCST90096922	35,653,391
Salad/raw vegetables consumption	422,542 European	GCST90096923	35,653,391
Added salt consumption	323,995 European	GCST90096924	35,653,391
Spirits consumption	118,477 European	GCST90096925	35,653,391
Tea consumption	434,171 European	GCST90096926	35,653,391
Vegetarianism	442,589 European	GCST90096927	35,653,391
Water consumption corrected for coffee	400,642 European	GCST90096928	35,653,391
Water consumption	445,799 European	GCST90096929	35,653,391

### Generic diet questionnaire

The genome associations of 29 food intake patterns were derived from a study by Pirastu et al.^23^. Analyses were conducted using data collected for the UK Biobank project (project no. 19655)^24^. A touchscreen dietary frequency questionnaire was used in the UK Biobank to assess dietary intakes and patterns^23^. The survey included questions regarding the frequency of consumption of specific foods and beverages.

All quantitative food and drink intake phenotypes were converted to weekly consumption; for example, drinking three cups of tea per day was converted to 21 cups per week. A semi-quantitative description^25^, such as never, two–four times per week, five–six times per week, and once or more per day, was converted to 0, 3, 5.5, and 7, respectively. Participants who chose not to answer or were not sure were excluded from the analysis.

All coffee traits were stratified by type (instant, ground, and decaffeinated) to account for differences in consumption patterns, such as cup size and caffeine concentration. We excluded participants who did not specify the type of coffee usually consumed.

Coffee consumption (any type of coffee, including unspecified) has a strong negative phenotypic correlation with water consumption; therefore, coffee consumption was treated as a covariate for water consumption. On the other hand, some semi-quantitative traits are not directly related to the amount or type of food or drink consumed. Non-dairy milk types (e.g., soy) were excluded from the calculation of milk fat content. The drink temperatures (very hot, hot, and warm) were converted to an arbitrary three-unit scale (3, 2, and 1, respectively). Individuals who did not consume hot drinks were excluded from the analysis. Supplementary Table S1 lists the number of samples used for each trait. Supplementary Table S2 provides a detailed description of the phenotypes.

### Genetic instrumental variable selection

MR analyses use SNPs as instrumental variables (IVs) to estimate the causal associations between exposures and outcomes^19^. MR analysis is based on three critical assumptions: (i) the exposure is strongly associated with the IVs, (ii) confounders for exposures and outcomes should not affect the IVs, and (iii) exposure is the only factor that mediates the IV-outcome associations^19^.

A linkage disequilibrium (LD) occurs when an allele in one locus is disproportionately coinherited with an allele in a different locus. Due to Mendel's second law of random assortment, using several genetic variants in LD between them may introduce biases in MR studies. As a first step, we determined whether the chosen independent genetic variants were significantly associated with each instrument for each exposure (*p* < 5 × 10^−8^). We applied clumping with R^2^ < 0.001 and a window size > 5,000 kb to avoid LD^26^. Averaging SNP-specific F-statistics was used to avoid weak IVs, and IVs with F values > 10 were considered strong^27,28^. A list of the selected SNPs is provided in Supplementary Table S3.

### Pleiotropy, heterogeneity, and sensitivity analysis

Genetic variants or genetic risk scores may be associated with other potential exposures or confounders; this phenomenon is known as pleiotropy. An estimate from an MR study could be unreliable if the genetic variants chosen are used in these circumstances^29^. We assessed the horizontal pleiotropy using MR-Egger regression, as indicated by the intercept^19^. A P-value < 0.05 indicates that the inverse variance-weighted (IVW) results might be invalid due to horizontal pleiotropy, and an MR Pleiotropy REsidual Sum and Outlier (MR-PRESSO) test should be conducted^30^. The degree of heterogeneity across all SNPs was evaluated using Cochran's Q statistic and leave-one-out analysis^31^. The results of the pleiotropy, heterogeneity, and sensitivity analyses are presented in Supplementary Tables S4, S5, and S6, respectively.

### MR analysis

Our primary analysis used the IVW method^32^; thus, all the variants were assumed to be valid IVs, providing the most precise results. The weighted median and MR-Egger regression methods were used in complementary analyses^33^. If the result obtained using the IVW method is significant (*P* < 0.05), it can be regarded as a positive result, even when the results from other methods are not significant, if the beta values of the other methods are in the same direction^34^. If horizontal pleiotropy was identified without heterogeneity, the MR-Egger method was selected; if heterogeneity was identified without pleiotropy, the weighted median method or the multiplicative random-effects inverse variance weighting (mre-IVW) method was used for the analysis. All the MR analyses were performed in R 4.2.1 (R Core Team [2022]. R: A language and environment for statistical computing. R Foundation for Statistical Computing, Vienna, Austria. URL https://www.R-project.org/), using the TwoSampleMR and Mendelian Randomization packages^35^. Supplementary Table S7 presents the MR estimates obtained using the different methods.

## Results

Our study included 26 types of food exposures, after excluding those without effective IVs (fortified wine consumption, spirit consumption, and vegetarianism). After a series of quality control steps, the SNPs chosen for each type of food ranged between 3 and 73. Among patients meeting the NMOSD diagnostic criteria, the ones with positive AQP4 antibodies were selected for the analysis. The F-statistic values were greater than the empirical threshold of 10, indicating that all SNPs were valid.

**Figure 2:**
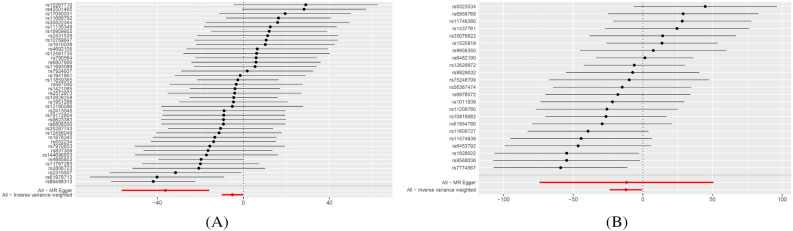
Forest plots for the food exposures. (**A**) Oily fish consumption. (**B**) Raw vegetable consumption.

Higher intakes of oily fish and raw vegetables were associated with a lower risk of AQP4-positive NMOSD. For oily fish consumption, the results of the IVW and MR-Egger analyses were statistically significant (odds ratio [OR] = 0.006, 95% confidence interval [CI] = 4.18 × 10^−5^–0.885, *p* = 0.045; OR = 1.78 × 10^−16^; 95% CI = 2.60 × 10^−25^–1.22 × 10^−7^; *p* = 0.001, respectively). The Cochran’s Q test showed no heterogeneity. However, pleiotropy was identified through the MR-Egger intercept analysis (*p* = 0.004), whereas the MR-PRESSO test did not show significant pleiotropy (*p* = 0.081). The leave-one-out studies were used for the sensitivity analysis and demonstrated no significant influence from individual studies. Considering the above findings, we selected the result from the MR-Egger method as the main one, as it was statistically significant. For raw vegetable consumption, the result from the IVW analysis was statistically significant (OR = 5.28 × 10^−6^; 95% CI = 4.67 × 10^−11^–0.598; *p* = 0.041). Heterogeneity and pleiotropy were not observed in this analysis. Leave-one-out studies were used for the sensitivity analysis and demonstrated no influence from individual studies. Overall, the consumption of oily fish and raw vegetables was associated with a lower risk of AQP4-positive NMOSD. Figure 2 shows the forest plots for exposure to oily fish and raw vegetables. The scatter plots, leave-one-out plots, and funnel plots are summarized in Supplementary Figs. S1 and S2. The results are summarized in Table 2.

**Table 2:** Results of the MR study testing causal association between oily fish/raw vegetables consumption and NMOSD.

Food intakes	Method	Number of SNPs	Beta	OR (95% CI)	P	P for heterogeneity test	P for MR-Egger intercept	P for MR-PRESSO (0 outliers)
Oily fish consumption	Inverse variance weighted	41	− 5.103	0.006 (4.18 × 10^−5^–0.885)	0.045	0.088	0.003	0.081
	MR Egger	41	− 36.263	1.78 × 10^−16^ (2.60 × 10^−25^–1.22 × 10^−7^)	0.001	0.33		
	Weighted median	41	− 4.340	0.013 (2.35 × 10^−5^–7.225)	0.178			
Raw vegetables consumption	Inverse variance weighted	23	− 12.151	5.28 × 10^−6^ (4.67 × 10^−11^–0.598)	0.041	0.118	0.988	0.133
	MR Egger	23	− 11.714	8.18 × 10^−6^ (6.65 × 10^−33^–1.01 × 10^22^)	0.716	0.092		
	Weighted median	23	− 9.133	0.0001 (1.36 × 10^−11^–858.05)	0.256			

The other 24 food intake exposures considered were not associated with the risk of AQP4-positive NMOSD (consumption of beef, beer or cider, spread on bread, bread, champagne or white wine, cheese, cooked vegetables, decaffeinated coffee, dried fruit, fresh fruit, ground coffee, instant coffee, lamb, non-oily fish, pork, poultry, processed meat, red wine, added salt, tea, poultry, and water, as well as water consumption corrected for coffee, drink temperature, and milk fat percentage). The intake differences between patients and controls were not significant using all methods.

## Discussion

Environmental factors are believed to influence significantly the risk and progression of NMOSD, particularly in women who are predominantly affected, with a female-to-male ratio of up to 9:1. Several putative risk factors have been suggested in previous studies, including ethnic or racial background, other autoimmune conditions, smoking, and infections^36^.

In susceptible individuals, certain foods may increase or decrease the risk of NMOSD^13,37^. We found that a higher consumption of oily fish was associated with a lower risk of AQP4-positive NMOSD. Eicosapentaenoic acid (EPA) and docosahexaenoic acid (DHA) are omega-3 polyunsaturated fatty acids (PUFAs) found in oily fish^38^. According to animal experiments and clinical intervention studies, omega-3 fatty acids exert anti-inflammatory effects via intracellular signaling pathways, transcription factor activity, and gene expression^39^. Several studies have examined the benefits of dietary supplements containing fish oils on inflammatory and autoimmune conditions, including ulcerative colitis, Crohn's disease, and rheumatoid arthritis^40^. In several placebo-controlled trials, fish oil has been shown to reduce the activity of chronic inflammatory diseases and the need for anti-inflammatory medications. According to experimental studies, the gut microbiota, omega-3 PUFAs, and the immune system play key roles in maintaining intestinal wall integrity^40^. Consequently, high oily fish consumption can improve the microflora profile and suppress the inflammatory process, and thus modification of the dietary patterns may reduce the susceptibility for NMOSD, according to the "gut-brain axis" hypothesis^12^.

On the other hand, raw vegetables have low energy density in addition to vitamins, fiber, folate, potassium, lignans, flavonoids, minerals, and other bioactive phytochemicals^41^. In a recent study, green salads were found to reduce the risk of all-cause mortality^42^. According to another study, raw vegetable consumption is inversely related to the level of DNA damage caused by oxidative stress^43^. Our statistical analyses revealed an association between high raw vegetable intake and low risk of AQP4-positive NMOSD. This finding can be explained by the abundant antioxidants found in raw vegetables. A high total antioxidant capacity (TAC) reduces the incidence of NMOSD and the risk of seropositivity^44^. As these disorders are astrocytopathy-mediated, patients with NMOSD are protected from free-radical damage by the antioxidants produced by astrocytes^45^. Astrocytes exert their effects in several ways, such as regulating the glutamate levels; nerve cells can be damaged by high levels of glutamate, a stimulatory neurotransmitter^44^. Astrocytes also produce glutathione (GSH), which protects neurons against oxidative damage. Finally, astrocytes activate the Nrf2–KEP1–ARE pathway in response to oxidative stress to protect neurons^46^. Thus, astrocytes may have a stronger activity against disturbances with the support of more antioxidants from the diet. We speculate that raw vegetables may reduce the risk of NMOSD through their anti-inflammatory properties, though few studies were conducted on this type of food.

## Conclusion and limitation

In conclusion, oily fish and raw vegetable consumption can decrease the risk of AQP4-positive NMOSD. The main strength of our study was the MR design, suitable for causal inference. RCTs on NMOSD are difficult to design and perform; therefore, an MR study may provide valuable insights into the risk of developing NMOSD associated with specific dietary components. Our study included 26 food types; some of the factors included, such as the consumption of processed psychoactive drinks, were not previously examined using MR. Therefore, future research on the relationship between food intake and disease risk may benefit from the results of this study.

However, the study also has some limitations. First, findings on several common types of NMOSD tend to have low statistical power, reducing their reliability^31^. The second limitation is that three of the food intake types considered had insufficiently effective IVs. Additionally, we examined single food items, whereas these elements may act synergistically or antagonistically in complex diets^47^. Furthermore, the existing literature on oily fish and vegetables and the associated risk of NMOSD is scarce, and this association should be explored with randomized controlled trials in the future. Researchers should examine various dietary patterns with MR studies to determine whether they affect the risk of developing NMOSD. We believe that several of the potential causal relationships described here have potential for further investigation^22^.

## Supplementary Information

Supplementary Information 1.

Supplementary Information 2.

Supplementary Information 3.

Supplementary Information 4.

Supplementary Information 5.

Supplementary Information 6.

Supplementary Information 7.

Supplementary Information 8.

## Data Availability

The human data used in this study are publicly available. All databases were obtained from the following website: GWAS Catalog (https://www.ebi.ac.uk/gwas/). The GWAS ID in Table 1 can be entered in the website to query and download the GWAS dataset used in this article.
