# Supplementary material for: Oily fish and raw vegetable consumption can decrease the risk of AQP4-positive neuromyelitis optica spectrum disorders: a Mendelian-randomization study
Source: Sci Rep. 2023 Jun 9;13:9372. doi: 10.1038/s41598-023-36372-1 (PMC10256733; doi:10.1038/s41598-023-36372-1)
Supplement: Supplementary file 8 — Supplementary Information 8. [file 41598_2023_36372_MOESM8_ESM.docx]

Supplementary Material

Oily fish and raw vegetable consumption can decrease the risk of AQP4-positive neuromyelitis optica spectrum disorders: a Mendelian-randomization study

Shengnan Wang^1^, Lin Pan^2^, Rui Wu^1†^, Yanqing Shao^1†^, Mengru Xue^1†^, Hao Zhu^3^, Wanwan Min^1^, Xiangyu Zheng^1^, Yekun Liang^4^, Mingqin Zhu^1, *^

**†These authors have contributed equally to this work and share third authorship**

*** Correspondence:** Corresponding Author: zhumingqin@jlu.edu.cn

# Supplementary Tables

**Supplementary Table S1** Number of samples used for the GWAS for each trait. The questionnaire column indicates from which questionnaire the item was taken. Proportion of sample indicates the proportion of samples used compared to the number of people who participated in the UK biobank at baseline (501,520).

**Supplementary Table S2** Description of the phenotypes: the table reports the description and coding of the phenotypes used for the GWAS analyisis. Phenotype, name of the phenotype; Question, question asked in the tochscreen questionnaire; Answer - Converted to, coding of the phenotype; Covariates, covariates used for the analysis, standard=age+sex; Transformation, transformation applied for normalising the trait.

**Supplementary Table S3.** Characteristics of the genetic instrument variables for the food takes in the Mendelian randomization study at the genome-wide significance level (P < 5 × 10^–8^).

**Supplementary Table S4.** MR analysis of 26 food intakes and AQP4-positive NMOSD risk.

**Supplementary Table S5.** Heterogeneity analysis of 26 food intakes and AQP4-positive NMOSD risk.

**Supplementary Table S6.** MR Egger intercept analysis of the association between food intakes and AQP4-positive NMOSD risk.

**Supplementary Table S7.** MR PRESSO analysis of the association between food intakes and AQP4-positive NMOSD risk.

## Supplementary Figures


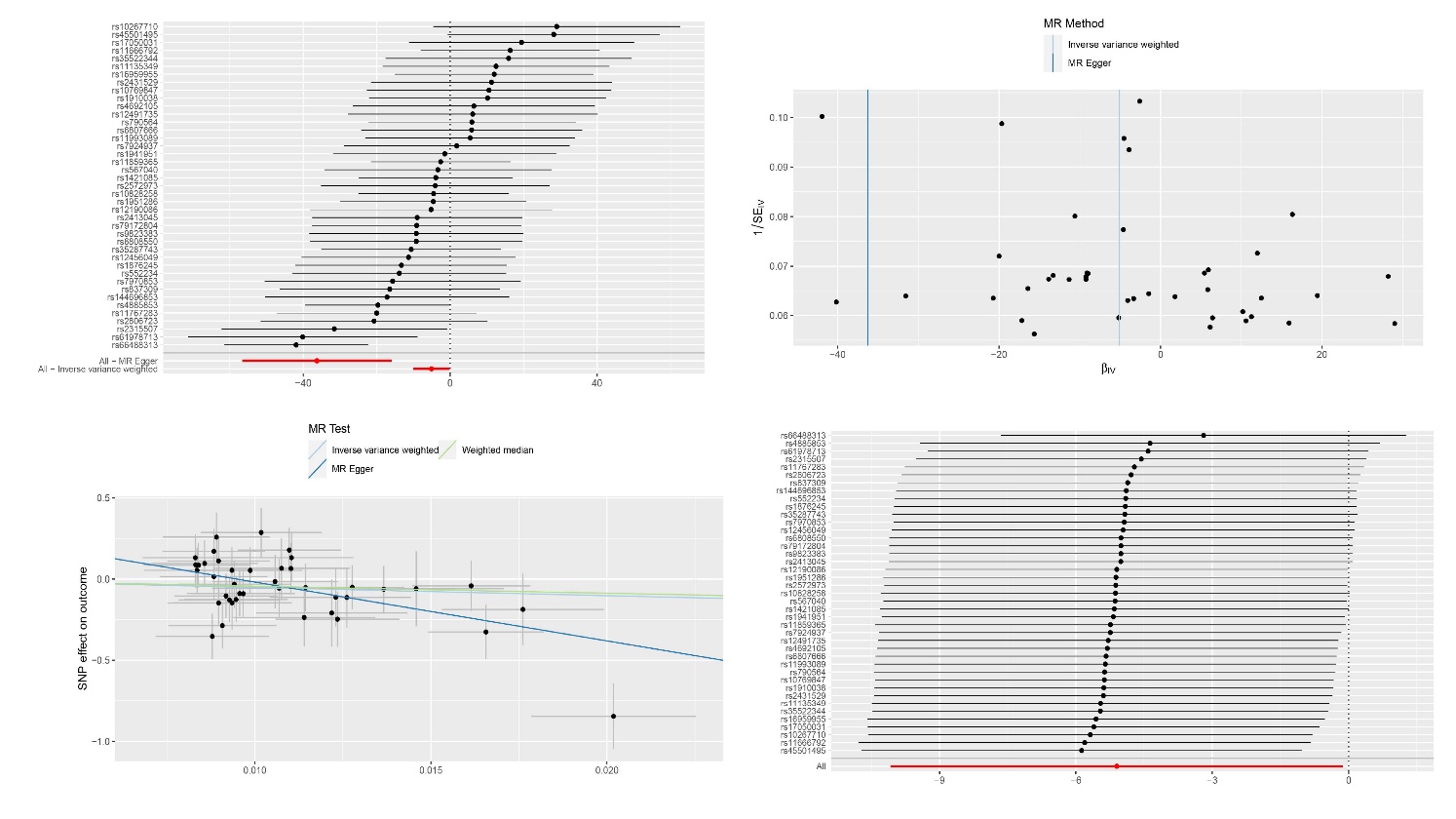


**Supplementary Figure 1.** Scatter plots (lower left), forest plots (upper left), funnel plots (upper right), and leave-one-out plots (lower right) for the exposure of oily fish consumption.


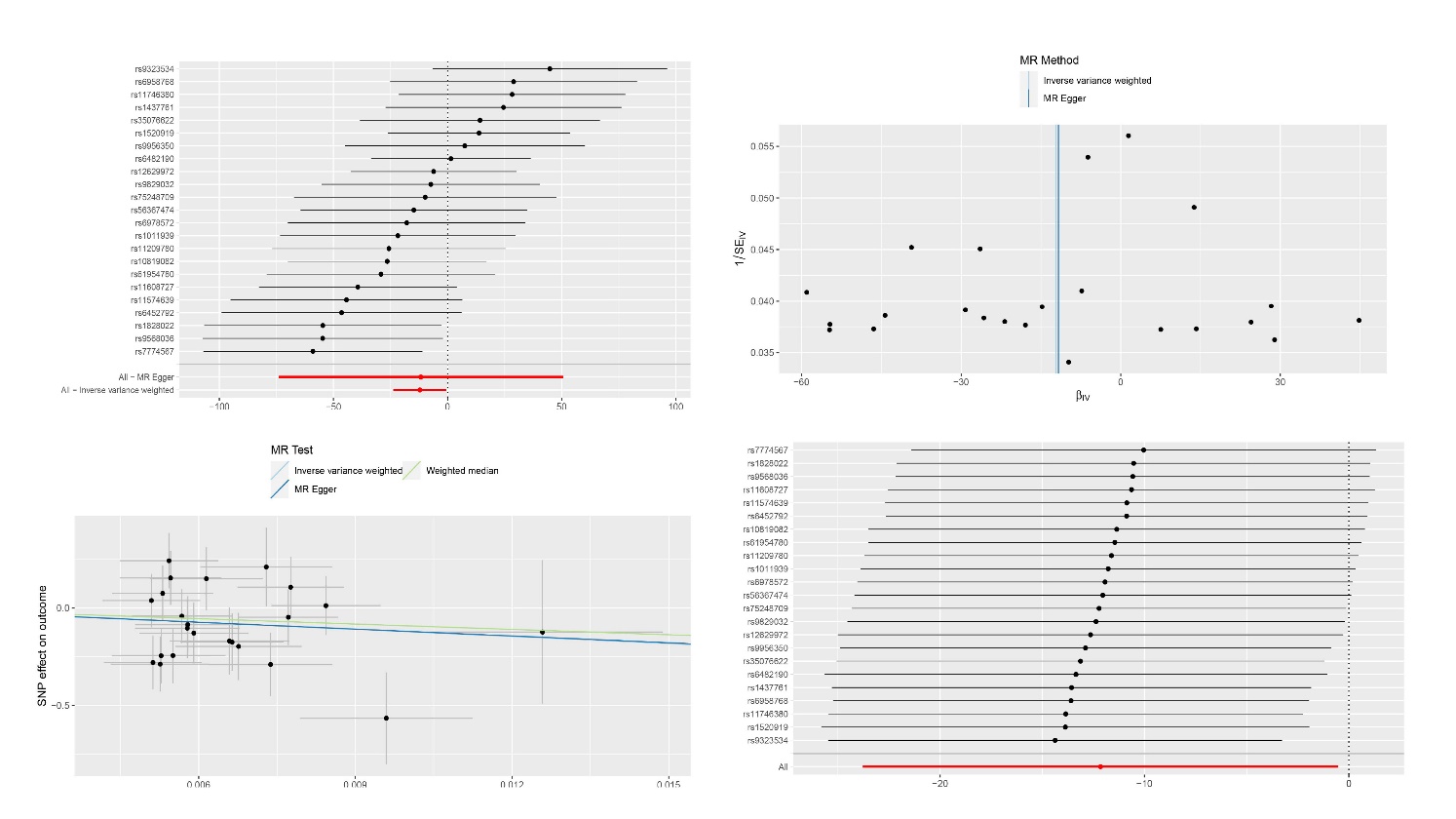


Supplementary Figure 2. Scatter plots (lower left), forest plots (upper left), funnel plots (upper right), and leave-one-out plots (lower right) for the exposure of raw vegetable consumption.
